# Improving person-centered occupational health care for workers with chronic health conditions: a feasibility study

**DOI:** 10.1186/s12909-023-04141-3

**Published:** 2023-04-07

**Authors:** Nina Zipfel, M. de Wit, N.C. Snippen, A.R. Bosma, C.T.J. Hulshof, A.G.E.M. de Boer, S.J. van der Burg-Vermeulen

**Affiliations:** 1 Department of Public and Occupational Health, Coronel Institute of Occupational Health, Amsterdam University Medical Center, University of Amsterdam, Amsterdam Public Health research institute, PO Box 22700, 1100 DE Amsterdam, The Netherlands; 2 Department of Public and Occupational Health, Amsterdam University Medical Center, VU University Amsterdam, Amsterdam Public Health research institute, Amsterdam, The Netherlands; 3 Department of Health Sciences, Community and Occupational Medicine, University of Groningen, University Medical Center Groningen, Groningen, The Netherlands; 4 Department of Public and Occupational Health, Amsterdam UMC, Coronel Institute of Occupational Health, PO Box 22700, 1100 DE Amsterdam, The Netherlands

**Keywords:** Occupational health, Implementation, Feasibility, Person-centered care, Education

## Abstract

**Background:**

Person-centered care is needed to effectively support workers with chronic health conditions. Person-centered care aims to provide care tailored to an individual person’s preferences, needs and values. To achieve this, a more active, supportive, and coaching role of occupational and insurance physicians is required. In previous research, two training programs and an e-learning training with accompanying tools that can be used in the context of person-centered occupational health care were developed to contribute to this changing role. The aim was to investigate the feasibility of the developed training programs and e-learning training to enhance the active, supportive, and coaching role of occupational and insurance physicians needed for person-centered occupational health care. Information about this is important to facilitate implementation of the tools and training into educational structures and occupational health practice.

**Methods:**

A qualitative study was conducted, with N = 29 semi-structured interviews with occupational physicians, insurance physicians, and representatives from occupational educational institutes. The aim was to elicit feasibility factors concerning the implementation, practicality and integration with regard to embedding the training programs and e-learning training in educational structures and the use of the tools and acquired knowledge and skills in occupational health care practice after following the trainings and e-learning training. Deductive analysis was conducted based on pre-selected focus areas for a feasibility study.

**Results:**

From an educational perspective, adapting the face-to-face training programs to online versions, good coordination with educational managers and train-the-trainer approaches were mentioned as facilitating factors for successful implementation. Participants underlined the importance of aligning the occupational physicians’ and insurance physicians’ competences with the educational content and attention for the costs concerning the facilitation of the trainings and e-learning training. From the professional perspective, factors concerning the content of the training and e-learning training, the use of actual cases from practice, as well as follow-up training sessions were reported. Professionals expressed good fit of the acquired skills into their consultation hour in practice.

**Conclusion:**

The developed training programs, e-learning training and accompanying tools were perceived feasible in terms of implementation, practicality, and integration by occupational physicians, insurance physicians and educational institutes.

**Supplementary Information:**

The online version contains supplementary material available at 10.1186/s12909-023-04141-3.

## Background

With the increase of the retirement age in most industrialized countries, the number of working-age people with a chronic condition has increased in recent years [1, 2]. In Europe, a quarter of the working population reports suffering from a chronic condition [3]. It is projected that the prevalence of chronic health conditions within the working population will continue to rise in the coming years [4]. The prevalence of chronic conditions in Europe has increased from 19 to 28% between 2010 and 2017 among people at working age [3]. Chronic conditions may have significant impact on work participation due to physical, emotional or social issues [2]. Significantly more workers with a chronic health condition leave paid employment due to unemployment, early retirement or receiving a disability pension compared to workers without a chronic condition [2, 5]. Return-to-work has been recognized as an important indicator for recovery of health and functioning and societal participation [6]. It is, therefore, important to facilitate return-to-work and promote work participation for people with a chronic condition [7].

Person-centered care has been acknowledged to positively influence people with a chronic health condition in terms of occupational performance and satisfaction [8, 9]. Person-centered care aims to provide care that is tailored to an individual person’s preferences, needs and values [10]. Person-centered care does not merely concern the individual person, but takes into account the entire person including the context and surroundings [11, 12]. The body of evidence surrounding person-centered care has increased over the past decade [13]. For instance, a systematic review found that person-centered care contributes to improved quality of care, self-efficacy, psychological and physical health status in patients with long-term chronic health conditions by using personalized care planning [14]. Additionally, to prevent prolonged sickness absence, person-centered care provided by clinicians was found to contribute to higher rates of return-to-work [15]. However, the implementation of person-centered care might be challenging by health care professionals due to the lack of clear professional guidelines, the lack of suitable personnel to deliver person-centered care and challenges in embedding person-centered care in the routine care process [16].

Within the field of occupational health care, person-centered guidance and work ability assessment by occupational physicians (OPs) and insurance physicians (IPs) has gained increasing recognition in recent years. Attention is growing towards enhancing self-control of workers with chronic conditions, understanding workers’ cognitions and perceptions regarding living with a chronic disease and work functioning, and involving significant others in supporting work participation [17–19]. In order to support the changing role of OPs and IPs to deliver more person-centered guidance and assessment, training programs and an e-learning training with accompanying tools have been developed [20–22]. The goal of the developed training programs and e-learning training with accompanying tools is to (1) increase self-control of workers with a chronic health condition by helping OPs to create a supportive work environment [20], (2) increase the ability of OPs and IPs to involve cognitions and perceptions in the guidance and assessment of workers [21], and (3) support OPs and IPs to involve significant others in the re-integration process [22].

To support better uptake of the developed training programs, e-learning training, and accompanying tools in practice, it is important to understand the factors affecting the implementation, practicality and integration [23]. The factors affecting the implementation include the degree, possibility and manner in which an intervention can be fully embedded in practice [23]. Practicality focusses on the aspects of the resources, time, commitment or a combination of those needed to deliver an intervention in practice [23]. Integration entails aspects of the changes needed in a system or environment to integrate an intervention into existing infrastructures [23]. In a previous study, determinants for the implementation of person-centered tools were identified [12]. The most important determinant was taking the needs of workers with a chronic health condition into account [12]. The results of this previous study give insight into the required focus for the implementation of person-centered tools in the field of occupational health care, but do not indicate whether it is feasible for professionals to apply the knowledge and skills gained in the training programs and tools in practice, and to embed the accompanying trainings and e-learning training in educational programs for OPs and IPs. Therefore, it is important to investigate the feasibility for the implementation from an educational perspective and professional perspective.

Investigation of the feasibility of developed training programs and e-learning training and accompanying tools is important as they lay the basis for broader application of research knowledge in practice [24]. The aim of this study was, therefore, to investigate the feasibility of the training programs and e-learning training with accompanying tools to enhance the supportive and coaching role of OPs and IPs for guidance and assessment of workers with chronic health conditions. The goal was to provide insight into how to facilitate implementation of the training programs and e-learning training into educational structures and practice.

## Methods

In order to investigate the feasibility, a qualitative study with semi-structured interviews was conducted. For this purpose, a qualitative research design was deemed most appropriate as it allows to gain a richer understanding of considerations for sustainable uptake and use of the previously developed training programs and e-learning training and accompanying person-centered tools in practice. The Consolidated Criteria for Reporting Qualitative research (COREQ) checklist was used to report on the interviews process [25].

### Research setting and context

In the Netherlands, two medical professions constitute the provision of occupational health care: OPs and IPs [26]. The OP is generally involved in the process of vocational support and return-to-work guidance for employees in the first two years of sick leave, as well as taking on preventive tasks such as promotion of healthy working conditions, improving sustainable employability, and early identification and treatment of occupational diseases. After the two-year sick leave period, a sick-listed employee is assessed for eligibility of a disability benefit by an IP. The IP assesses the functional abilities, limitations, and consequences for a person’s work ability. In case of partial work disability the IP can refer the employee for return to work interventions or support. For an employee who has no (longer) an employer, the IP takes on the role of an OP to guide and support return to work in the first two years of sick leave. For both professions after the basic medical education, they receive a four year postgraduate training at dedicated non-profit educational institutes [27, 28]. The resident training to become either an OP or IP is offered at either the Netherlands School of Public & Occupational Health or at the social medicine education department of the Radboud university medical center. In the Netherlands, OPs and IPs, when officially registered as practicing physicians, need to follow continuing professional education. The continuing professional education can be offered by either non-profit educational institutes, the educational department of the Dutch Social Security agency or private suppliers.

The development and evaluation of the trainings and e-learning training was part of a larger Dutch research program aimed at contributing to improved worker-focused occupational health care. The research program consists of three research projects which were conducted in parallel to improve the supporting role of OPs and IPs in occupational health care for workers with chronic health conditions. As aforementioned, the topics covered in the training programs and e-learning training were respectively: creating a supportive work environment to enhance self-control of workers [20], involving cognitions and perceptions of workers in guidance and assessment [21], and involving significant others in the re-integration process [22]. More information on the content of the training programs, e-learning training and accompanying tools can be found in Table 1. The training program on creating a supportive work environment was targeted only at OPs. In the Dutch context IPs generally do not stand in contact with an employer and therefore only OPs have the possibility to directly influence the work environment. The three projects previously evaluated the developed training programs and e-learning training in terms of acquired knowledge and skills, and satisfaction with the trainings and e-learning training. With regard to the training on creating a supportive work environment to enhance self-control of workers, participants were asked about their satisfaction with the training and a process evaluation was conducted identifying possible barriers and facilitators for broader implementation [20]. With regard to the training on involving workers’ cognitions and perceptions in guidance and assessment, the effect of the training program on the ability of OPs and IPs to identify workers’ cognitions and perceptions to recommend evidence-based interventions to address at limiting cognitions and perceptions of workers was studied in a randomized controlled trial [29]. In addition, the satisfaction with the training program was evaluated by means of a questionnaire and some feasibility aspects were evaluated during interviews [21]. With regard to the e-learning training on involving significant others in the re-integration process, a randomized controlled trial was conducted to evaluate the efficacy for improving OPs’ and IPs’ knowledge, attitudes, and self-efficacy to involve significant others in the return-to-work process. Furthermore, the OPs’ and IPs’ responses to and satisfaction with the e-learning training were explored [22].

**Table 1:** Description of the developed person-centered tools and accompanying trainings and e-learning training

The tool	Description of the tool	Description of the educational form and content
**(1) Strengthening self-control of workers with chronic health conditions**	A training was developed for OPs to guide organizations with creating a supportive work environment for workers with a chronic health condition, in order for these workers to exert self-control. In the training, OPs learn to become a process leader and guide organizations to apply the Participatory Approach (PA). The goal of the PA is to identify and prioritize existing problems within an organization and to identify and implement solutions for strengthening self-control of workers with a chronic health condition.	The education is offered in the form of a face-to-face training in two parts. The first training session is organized as a half-day educational program in which a combination of theory and practical application are taught to the OPs. The second meeting consisted of a peer review meeting in which experiences with applying the PA in practice were shared among OPs.
**(2) Involving person-related factors (cognitions and perceptions) in occupational health care**	Based on previously conducted research, a training was developed to teach both OPs and IPs how to involve the worker’s cognitions and perceptions in the occupational health management and work disability assessment of workers with a chronic health condition. The participants acquire knowledge of ten cognitions and perceptions identified as most important for work participation, learn how to obtain information on those cognitions and perceptions during consultation and learn how to intervene on limiting cognitions and perceptions. During the training a conversation tool is presented to the OPs and IPs to support the use of the acquired skills in daily practice.	The training program is organized as a half-day program with a duration of 4.5 h. The training consists of classical presentations and different practical individual and group exercises, including case learning. Eligible trainers are OPs and IPs with extensive experience in occupational health care.
**(3) E-learning training “Training for Occupational health physicians To Involve Significant others”**	The e-learning training “Training for Occupational health physicians To Involve Significant others” was developed to educate Ops and IPs on how they can best address the role of significant others and manage their involvement in the return-to-work process of sick-listed workers with chronic diseases. The e-learning training was accompanied by a conversation tool, which included: (1) a reference book containing an overview of the key messages and practical advice from the e-learning training, (2) validated questionnaires with which OPs could gain insight into illness perceptions and coping of workers and their significant others, (3) a conversation leaflet that was developed to facilitate communication between workers and significant others, and (4) ten leaflets about different chronic diseases that were developed to promote adequate illness perceptions of workers and significant others.	The e-learning training can be followed at the participants’ own pace and consists of five parts focused on delivering essential knowledge with regard to involving significant others and translating that knowledge into practical skills. The first four parts included interactive components, such as videos or vignettes in combination with multiple-choice questions. The fifth part consisted of a summary of key messages and best-practice recommendations from the first four parts. Participants could access the e-learning training and download the accompanying conversation tool in an online learning environment to which they had unlimited access.

A. OPs: Occupational physicians, IPs: Insurance physicians

The study was considered not to fall under the Dutch Medical Research Involving Human Subjects Act (WMO) as approved by the local ethics committee of the Amsterdam UMC (Reference number: W19_949#20.012 and W20_024#20.050). The study was conducted according to the guidelines laid down in the Declaration of Helsinki.

### Data collection and participants

Qualitative data on the implementation, practicality and integration of the developed trainings and e-learning training and accompanying person-centered tools were collected based on the feasibility study design recommendations by Bowen et al. (2009) [23]. Following the definitions formulated by Bowen et al., the following feasibility aspects were examined: implementation, practicality and integration [23]. Those focus areas were deemed most important to facilitate future implementation into practice and education. For the data collection, semi-structured interview guides (Additional file 1) were used based on the selected focus areas from Bowen et al. (2009) [23].

To gain insight into these focus areas, individual interviews were held from two perspectives: (1) an educational perspective to gain insight into implementation strategies for uptake of the training and e-learning training in existing educational structures and (2) a professional perspective [20–22]. For the educational perspective, representatives from different educational institutions involved medical educational training for OPs and IPs were interviewed (N = 5), including resident trainers from the specialized institutions (Netherlands School of Public & Occupational Health and the social medicine education department of the Radboud university medical center) and the Dutch Social Security Agency. The educational experts were not previously involved in providing the training or e-learning training. As the start of the interview they received a short introduction with the learning goals per training and e-learning. With regard to the e-learning training, the educational experts were given access to the entire e-learning prior to the interviews. The interview questions for the educational perspective were the same for all three projects as the goal was to explore their opinions in general. For the professional perspective, participants from the previous evaluation studies of the three projects were included: project (1) N = 7; project (2) N = 11; project (3) N = 6. The participants were included from the sample of participants that were involved in one of the projects. This means that each interviewee participated in one of the projects previously and had thus only received one of the developed trainings or e-learning training. For the professional perspective, interview questions were each adapted per project to fit the goals and set-up of the specific training program or e-learning training. All participants were included by means of purposive sampling from either the network of the research program or the participant list from the prior evaluation studies of the three projects. All professionals that participated in the previously conducted pilot evaluations were invited. From project (1) 70% (N = 7 from N = 10), from project (2) 19% (N = 11 from N = 57) and from project (3) 9% (N = 6 from N = 62) of the invited professionals agreed to participate in this follow-up study. All participants were invited to participate via e-mail and gave written informed consent upon participation. The interviews were conducted by a minimum of one author, audio recorded and transcribed in Dutch. The interviews for project (1) were conducted in 2019 and 2020 (by AB) as phone or video-call interviews, for project (2) in 2020 (by NZ and MdW) as phone interviews, for project (3) in 2021 (by NZ and NS) as online interviews by video-call. All interviews from the professional perspective lasted approximately 30 min. The interviews from the educational perspective were conducted in 2020 by NZ and SvdB-B and lasted approximately one hour. All researchers are experienced in conducting qualitative research. Authors NZ, MdW, AB and NS are no occupational health professionals. The other authors are experts from the field of occupational health. For the interviews from the professional perspective, authors AB and MdW had earlier contact with the participants in the context of the evaluation studies. Author NZ and NS did not have an established relationship with participants prior to the interviews.

### Data analysis

The interviews were initially transcribed verbatim and analyzed for both perspectives (educational perspective and professional perspective) per training program and e-learning training with accompanying person-centered tool. Each perspective was analyzed for the three focus areas separately as recommended by Bowen et al. [23]. They recommend several sample outcomes of interest for the three focus areas. For implementation the following outcome was included in this study: factors affecting implementation ease or difficulty. For practicality the goal was to gain insight into the following outcomes: positive/negative effects on target participants, ability of participants to carry out intervention and/or educational activities, and cost analysis. For the integration the following outcomes were included: perceived fit with (practice and/or educational) infrastructure, perceived sustainability, and costs to organization and policy bodies. For the data analysis, the following steps were followed: organizing the data, reading and memoing to become familiarized with the data, and forming codes into feasibility factors organized into the three pre-chosen outcomes. In the first step of the analysis open coding based on content analysis was applied to identify feasibility factors, which was followed by deductive coding with thematic analysis based on the focus areas and pre-determined outcomes. After the analysis per perspective for the three projects separately, additional cross-project analysis was conducted to identify feasibility factors per category that appeared across the three projects and those that are unique to the projects. The goal of this analysis was to find overarching feasibility factors to facilitate implementation of the training programs and e-learning training into education structures and practice. All semi-structured interviews were analyzed by two independent researchers (NZ and a research assistant) for project (1), NZ and MdW for project (2), except for the professional perspective from the third project which was analyzed by one researcher (NZ) and checked by a second researcher (NS). Additionally, a third researcher (SvdB-V) checked all codes. Analyses of the interviews were performed in MAXQDA 2020.

## Results

The results are presented per perspective for (1) the educational perspective concerning the feasibility of embedding the developed trainings and e-learning training in existing educational structures, and (2) the professional perspective for the feasibility of using and applying the knowledge and tools in practice. For readability, only the cross-project feasibility factors are presented (Table 2). In case no cross-project feasibility factors were found only the most important results will be presented in the result section below. The detailed results per tool from both perspectives can be found in Additional file 2.

**Table 2:** Summary of the cross-project analysis of feasibility factors per Bowen et al. outcome from the educational perspective

Bowen et al. focus area	Outcome	Category	Feasibility factor	Project
1) Feasibility factors from an educational perspective				
Implementation	Factors affecting implementation ease or difficulty	Factors concerning the trainings and e-learning	Offering an online version of the training	(1), (2), (3)
		Factors concerning the organization of the trainings and e-learning	Coordination with the educational managers of the educational institutions	(1), (2)
			Offer a train-the-the trainer course	(1), (2)
			Make arrangements regarding the ownership of the training and e-learning	(2), (3)
			Coordinate with executive education manager	(1), (2)
Practicality	Cost analysis	Costs for the organization of the trainings and e-learning	Rental costs of training facility	(1), (2)
			Costs for accreditation of training and e-learning	(1), (3)
Integration	Perceived fit with educational infrastructure	Suitability within educational structures	Not suitable for core curriculum of postgraduate medical training for OP/IP	(1), (3)
			Added-value of the training and e-learning is evident	(1), (3)
		Fit within the curriculum	No unlimited place to embed new trainings in current curriculum	(2), (3)

### 1) Feasibility factors from an educational perspective

For the educational perspective, five interviews were held with trainers from educational institutes (Table 3). Two females and three males with a mean age of 54.4 years of age participated. All participants had insight into all available material of the training programs and e-learning training and received a description by the researchers (NZ and SvdB-V). For the analysis of the Bowen et al. outcomes, the cross-project analysis yielded several feasibility factors which are presented below (Table 3). However, these presented factors are not exhaustive and the detailed results per training program and e-learning training with accompanying tools can be found in Additional file 2.

**Table 3:** Demographic characteristics of participants from an educational perspective

Participant	Occupation during interview	Experience in current function (years)
P12	OP and institute trainer	20
P13	OP and institute trainer	unknown
P14	IP and institute trainer	27
P15	IP and institute trainer	16
P16	OP and institute trainer	20

A. OP: Occupational physician, IP: Insurance physician

#### Implementation

Different ‘factors affecting implementation ease or difficulty’ concerning the training and e-learning training, organization of the education, dissemination and personal factors were identified (Table 3). With regard to the training programs, to have an online version available was mentioned by the educational experts as a way to support implementation across all three projects. Specific factors related to the e-learning training included a ‘check if the e-learning training was completed’ and ‘the combination of educational forms towards blended learning’ (Additional file 2), as one trainer mentioned:

*P12: “What we ultimately want to achieve is a form of blended learning in which physical and online education and e-learnings are all integrated into a complete package. And the great thing about this is, that they [students] can do a lot on their own, in their own time.”*

The ‘check if the e-learning training was completed’ was mentioned by some participants as important. As this is already part of the current e-learning training, they felt this should stay in place as is. Moreover, it was mentioned that ‘sufficient interaction’ between participants during the trainings needs to be ensured for successful implementation (project 2) (Additional file 2). For the organization of the trainings, educational experts mentioned that good ‘coordination with the educational managers of the educational institutions’ is needed to ensure better implementation into educational structures (as to project 1 and 2). For both face-to-face training programs (project 1 and 2), ‘a train-the-trainer approach’ was indicated as a factor to support better implementation into educational structures, as well as to ‘make arrangements regarding the ownership of the training and e-learning’:

*P16: “[…] on my practical experience, for example, […] an organization takes ownership and then it [the training] comes behind a pay-roll.”*

Across the three projects no overarching feasibility factor related to the dissemination was found. A specific factor mentioned to enhance better dissemination of the e-learning training was ‘the use of role models or frontrunners’(Additional file 2):

*P14: “Yes, my tip is […], implementations become successful because you have someone who is going to promote the product and actually implements it and just does it. Someone that sells it. That’s what it really comes down to.”*

With regard to personal factors of educational experts that may hinder implementation no feasibility factor was found across all projects. However, it was specified that it is important to be aware that educational experts may be reluctant when it comes to incorporating new training materials from a third party (i.e., researchers) in the curriculum, which underlines the need to create good support from within the educational institutions (project 3) (Additional file 2).

#### Practicality

Across the projects no common feasibility factors were found for the outcome ‘positive/negative effects on target participants’ and ‘ability of participants to carry out intervention activities’. For project (1), it was indicated that with respect to the practicality outcome ‘positive/negative effects on target participants’, the added-value of the training for OPs needs to be clearly explained to enhance external and internal motivation to follow the training (Additional file 2). As to the ‘ability of participants to carry out educational activities’, some participants from project (2) mentioned the importance to match the educational content with the level of pre-existing knowledge and skills of participants as to offering the trainings to registered OPs and IPs or to resident doctors in training (Additional file 2). Furthermore, some participants from project (2) stressed the ‘difficulty to translate knowledge and skills into own practice’ which might hinder the practical uptake of the trainings into practice of the OPs and IPs:

*P13: “What we notice in the training groups is that at least some of the participants say at the end of the day: ‘it was very useful, but I don’t see myself doing it [applying the knowledge in practice] yet’. And, therefore, they have a difficulty in translating it into practice, into their own practice.”*

With respect to the costs of offering the trainings and e-learning training, some participants mentioned the following important factors to take into account: ‘costs for use of training facility e.g. rental costs’ (project 1 and 2) and ‘costs for accreditation’ of the training programs and e-learning training (project 1 and 3) (Table 3).

#### Integration

For the integration of both training programs and the e-learning training, the ‘perceived fit with educational infrastructure’ was evaluated (Table 3). For project (1) and (3) in terms of suitability within educational structures, participants mentioned that the training and e-learning training was ‘not suitable for the core curriculum of postgraduate medical training for OPs and IPs’ even though the ‘added-value of the training and e-learning training is evident’. Remarks were made regarding the integration in the current curriculum of the postgraduate medical training for OPs and IPs as to that there is ‘no unlimited place to embed new trainings in the current curriculum’ (project 2 and 3). Especially with respect to the training on strengthening self-control of workers with chronic health conditions (project 1), participants stressed that it can best be integrated towards the end of postgraduate medical training for OPs and IPs due the level of required pre-existing knowledge and skills, and they indicated that it predominantly fits the profession of OPs instead of IPs as the training is targeted at OPs (Additional file 2). For project (3), in terms of the outcome ‘perceived sustainability’, the ‘continuity after the research project ends’ was mentioned stressing the importance of continuity after an experimental setting of testing training programs.

### 2) Feasibility factors from the professional perspective on embedding the trainings and tools in educational structures and practice of OPs and IPs

For the professional perspective, a total of N = 24 semi-structured interviews were conducted. Participants were N = 18 OPs and N = 6 IPs who participated in the previous evaluation studies [20–22] (see Table 4). In total N = 13 males participated and N = 11 females. The mean age of participants for project (1) are unknown. The mean age of participants of project (2) was 48.5 years of age and for project (3) 52.3 years of age. Years of experience in current work function was unknown for project (1). The professionals gave input on their practical experiences after attending the trainings or following the e-learning training, but also gave input regarding the possibilities for the implementation of the trainings and e-learning training in educational structures from the perspective of a potential receiver of the training and e-learning training.

**Table 4:** Demographic variables of participants from a professional perspective

Participant	Participated pilot training/e-learning training	Occupation during interview	Experience in current function (years)
P18	Project (1)	OP^A^	Unknown
P19	Project (1)	OP	Unknown
P20	Project (1)	OP	Unknown
P21	Project (1)	OP	Unknown
P31	Project (1)	OP	Unknown
P32	Project (1)	OP	Unknown
P24	Project (1)	OP	Unknown
P1	Project (2)	OP	25
P2	Project (2)	IP^A^	6
P3	Project (2)	OP	30
P4	Project (2)	IP	10
P5	Project (2)	IP in training	3
P6	Project (2)	OP in training	3
P7	Project (2)	OP	29
P8	Project (2)	IP	0
P9	Project (2)	IP	41
P10	Project (2)	IP in training	2
P11	Project (2)	OP	20
P25	Project (3)	OP	> 20
P26	Project (3)	OP in training	2
P27	Project (3)	OP	> 20
P28	Project (3)	OP	25
P29	Project (3)	OP and IP	> 20
P30	Project (3)	OP	15

A. OP: Occupational physician, IP: Insurance physician

For the analysis across the three projects from a professional perspective, common feasibility factors were only found for the feasibility aspect of implementation which entails the Bowen et al. outcome ‘factors affecting implementation ease or difficulty’ (Table 5). For the practicality and integration project-specific feasibility factors were found (Additional file 2).

**Table 5:** Summary of the cross-project analysis of feasibility factors per Bowen et al. outcome from the professional perspective

Bowen et al. focus area	Outcome	Category	Feasibility factor	Project
2) Feasibility factors from the professional perspective				
Implementation	Factors affecting implementation ease or difficulty	Factors concerning the training and e-learning	Use of actual cases from practice	(1), (2)
			Need for periodic reminder or refresher about the topic	(1), (3)
		Factors related to the tools	Use of desk manual/summary as handy memory aid	(2), (3)
		Factors related to the dissemination	Embed in medical guideline	(1), (3)

#### Implementation

In terms of implementation, the outcome ‘factors affecting implementation ease or difficulty’ was evaluated. Based on the semi-structured interviews, the following factors were identified by professionals: personal factors;factors related to training programs and e-learning training and the tools; factors related to the organization of the training or e-learning training, and factors related to the dissemination were identified by the professionals. Factors concerning the training included the ‘use of actual cases from practice’ (project 1 and 2) and the ‘need for a periodic reminder or refresher about the topic’ (project 1 and 3). Specifically for project (1) on strengthening self-control, participants mentioned the necessity for ‘matching the training content with the needs within organizations’ to apply the Participatory Approach, targets organizations where OPs are involved in policy setting regarding support of workers with a chronic health condition (Additional file 2). Therefore, OPs need to be involved more in policy setting within organizations. With regard to the organization of the training and e-learning training no common feasibility factors were found across all three projects. For project (1) it was mentioned to ‘involve the researcher of the project’ when offering the training program. The ‘use of a desk manual or summary as a handy memory aid’ was mentioned for the use of the tools with regarding project (2) and 3).

In terms of dissemination factors for project (1) and (3), the suggestion was made to ‘embed the knowledge into guidelines’ (Table 5). In terms of the personal factors, no across-project factors were found, but project-specific factors important to consider included, for example, ‘sufficient time’ during the consultation to apply the acquired knowledge and skills (project 2) (Additional file 2):

*P3: “Well, you always have to take the time yourself as an OP [during consultation].[…] I can take that [time] by doing longer consultation hours, that’s not the problem. (project 2)*

For project (1) specific prerequisites for the implementation were mentioned including: organizational support, creating a sense of urgency, and creating recognition of importance for the target group. Concerning impeding factors for the implementation of project (1), the influence of the size and structure of the organization where the tool shall be applied is essential with higher chances for successful implementation in organizations with sufficient resources to invest in workplace improvement. With respect to the suitability, it was also mentioned that project (1) can be easier implemented among self-employed OPs as they have more freedom to make changes to their way of working (Additional file 2).

#### Practicality

For the outcomes on practicality, concerning the practical uptake of the developed training programs, e-learning training and accompanying tools into practice, the ‘ability of participants to carry out intervention activities’ (e.g. use of conversation tool and supporting material), ‘positive/negative effects on target participants’ and ‘cost analysis’ based on the suggested outcomes by Bowen et al. were evaluated from the professional perspective. As to the Bowen outcome ‘positive/negative effects on target participants’ feasibility factors were only found for project (3) and included the ‘added value for participants’ of the skills they acquire during the e-learning training (Additional file 2). No across-project feasibility factors were found for the outcome ‘ability of participants to carry out intervention activities’. As to project (2) about involving person-related factors, participants mentioned ‘not knowing it [the list of cognitions and perceptions] by heart after the training’ as a restrain for applying the knowledge in practice for the Bowen outcome ‘ability of participants to carry out intervention activities’ (Additional file 2):

*P4: “You know, the moment that you are in a consultation hour, you will no longer have all those sample questions in front of you. So, you have to do it a bit by heart. Apparently, the material has not yet sunk in so that I know it all by heart, so to speak.” (project 2)*.

For project (3), the participants stressed that the theoretical knowledge increased their awareness in practice as it increased their sense of importance as to involving significant others during the consultation with a worker with a chronic health condition (Additional file 2). Regarding the practical application of the tool developed in project 1), the participants mentioned the need for ‘more support for unexperienced OPs’.

Also no common feasibility factor was found for the Bowen outcome ‘cost analysis’. As to project (1), in terms of the ‘costs analysis’, one participant expressed feelings of ‘uncertainty about cost-effectiveness of the tool’:

*P20: “Well it also didn’t work on a small-scale, but maybe on a large-scale it would have succeeded, because then the time investment, so the total investment is the same, but perhaps much more profitable for an organization.” (project 1)*.

#### Integration

Professionals reported on the outcome ‘perceived fit with infrastructure’ for each of the three projects, but no common feasibility factor was found. However, project-specific factors were mentioned (Additional file 2). For project (1) a category on the integration at an organizational level was identified and included the following feasibility factors: ‘include in the organization’s annual plan’ and the ‘degree of professional flexibility of the OP’ which may contribute to better integration of the training program. Only for project (1) the outcome ‘perceived sustainability’ was mentioned as to no continuity of implementing the knowledge in practice after following the training can be guaranteed which is needed for the success of the training.

## Discussion

The aim of this study was to investigate the feasibility in terms of factors concerning the implementation, practicality and integration of trainings and e-learning training with accompanying tools for improved guidance and support for workers with a chronic health condition. The results show that the training programs, e-learning training and tools were seen as feasible from an educational as well as professional perspective when considering the mentioned factors. In order to contribute to successful implementation in educational structures and embedding the developed training programs, e-learning training and tools into the practice of occupational health care several factors were mentioned by the participants across the three projects, including adaptation to online versions of the face-to-face trainings, train-the-trainer approaches to facilitate correct delivery of the face-to-face trainings, costs concerning the implementation of the trainings and e-learning training, the use of actual cases from practice during the trainings and e-learning training, and follow-up trainings in the form of blended learning. By involving the researchers and actual users of the developed training programs and accompanying tools in an early state during implementation optimal fit into practice is warranted.

The importance of conducting a feasibility study has been recognized earlier [23, 30–32]. By evaluating three major focus areas for assessing the feasibility, our study aimed to provide insight into the factors concerning the implementation, practicality, and integration of person-centered and organization centered tools. Especially in occupational health care where not one single intervention may have an impact on workers feasibility studies are essential [31]. While previous studies on the training programs and e-learning training with accompanying tools also evaluated aspects of acquired knowledge and skills, and satisfaction [20–22], the current evaluation focused specifically on broader educational and practical aspects of the implementation. Another previous study investigated the determinants for the implementation of person-centered tools from the users’ perspective for the suitability of the tools for the target group [12]. This study identified that taking into account the individual needs and wishes of workers would support successful implementation of the tools in practice during consultation with an OP or IP [12]. However, that study was conducted during the early-stages of the development of the tools to support co-creation with the end-users, but did not investigate practical aspects of delivering the training programs and e-learning training. Evaluation of the feasibility of a training in occupational health care has been performed previously to enhance guideline use in occupational health care [33]. Similar to the current study, they identified time, organizational constraints and financial aspects as barriers for implementation [33]. To transfer knowledge and skills for guideline use, training has been found an effective method to facilitate uptake [34]. Our study also showed that offering training is a suitable way, but particularly effective if combined with online education (i.e., blended learning). Blended learning is the combination of face-to-face education and technology-mediated instruction [35]. The current study found that only technology-mediated instruction like the e-learning training for project 3) can be helpful to learn the theory, but to translate the skills into practice, it would require accompanying face-to-face education. Nevertheless, e-learning has advantages over face to face training, for example, being able to follow the education at the student’s own pace [35]. The argument to add more actual cases from practice during the trainings and e-learning training as reported by participants in our study was also confirmed by previous studies as it improves integration of new skills with current knowledge of professionals [33, 36]. Moreover, a train-the-trainer model as mentioned by participants in the current study could potentially enhance correct delivery of the training programs. A train-the-trainer model for the studied training programs and e-learning training could include training sessions from the involved researchers offered to potential trainers in practice. Train-the-trainer models may have the potential to contribute to more sustainable programs due to the involvement of trainers in early stages of a program [37]. Future research should establish a suitable model for training potentials trainers on the training programs and e-learning training. In order to integrate the training programs and e-learning training into existing educational structures and practical use of acquired knowledge and accompanying tools for OPs and IPs should address these factors for more successful uptake.

### Limitations

For all the respective projects, the goal was to investigate the factors concerning the three focus areas (implementation, practicality, and integration) of Bowen et al. from both the educational, as well as professional perspective. However, the interviews for project (1) used a different interview approach for the professional perspective due to convenience. In the context of that project, interviews were held with the goal of exploring barriers and facilitators for implementation of the tool and identifying possible points of improvement for the training, but without the specific goal of asking about implementation, practicality, and integration factors as in the other projects. This might have led to project-specific factors going unrecognized as the questions were not as specific during the interviews. Moreover, in the second step of the analysis a cross-project analysis was conducted to find overarching feasibility factors across the three projects. The interviews for the educational perspective were all held based on the same interview guide, which might have influenced the results of finding feasibility factors for all three focus areas (implementation, practicality, and integration) compared to the professional perspective where only factors on the implementation were found. The different structure of interviews and diverse character of the training programs and e-learning training may have influenced the result of finding rather project-specific factors instead of overarching cross-project factors. Therefore, the results from the professional perspective need to be considered per training program and e-learning training. However, the goal of the cross-project analysis was not to stress importance of certain feasibility factors, but to illustrate commonalities and differences between the three projects. Furthermore, the factors concerning the educational structure in the Netherlands may not be generalizable to other contexts. The educational structure in the Netherlands is quite unique for postgraduate medical training to become occupational health care professionals. Yet, the factors identified in this study may not be limited to a certain group of medical education, but may be viewed in a wider context for general factors identified as costs, for example. Moreover, the selection of participants might have impacted the results as participation was voluntary and the picture of the most motivated trainers and occupational health professionals may be portrayed which may have led to more favorable and positive results.

### Recommendations for future research and practice

The current study provides insight into possible factors affecting the implementation, practicality, and integration of the training programs, e-learning training and accompanying tools in educational structures and occupational health care practice. However, due to the explorative qualitative design of this study specific goal-setting may be challenging and not all factors are feasible to be tackled for future implementation. Therefore, future prioritization of the most important factors would help for the formulation of tailored implementation strategies and translation into workable implementation strategies. Furthermore, to evaluate the effect of the training and e-learning training on certain outcome measures, as for example improved person-centered care or improved participation, further study should be conducted on a larger scale. The current feasibility study provides a basis for future larger studies aimed at improving person-centered occupational health care. For practical uptake into existing educational structures, the current trainings and e-learning training need to be adapted taking into account the identified factors. Specifically, adaptation focusing on the factors as offering online versions of the training programs and offering a train-the-trainer course might be promising for broader reach and to support provision of the training programs and e-learning training as intended. To safeguard use of the acquired knowledge in practice, the training programs and e-learning training need to be structurally integrated into existing educational structures. Our study found that it is challenging to fit new trainings into the existing curriculum of OPs and IPs, but since the added-value of acquired skills in the practice of OPs and IPs, embedding these trainings as elective courses or as refresher courses might be a suitable approach for the integration of the training programs and e-learning training. For sustainable implementation and attracting professionals to follow the training, we recommend using case-based learning with more actual cases from the practice of the professionals and incorporating periodic reminders, by means of e-mails for example, to stimulate application of the acquired skills in practice. Currently, follow-up studies with pilot implementations in occupational health practice are being set-up.

## Conclusion

In this study, the feasibility of the developed trainings and e-learning training and accompanying tools were evaluated and perceived as feasible in terms of implementation, practicality, and integration. In addition, possible barriers to the implementation and practical use were identified. All three tools were perceived as valuable with adaptions as proposed in the current study. Future larger-scale implementation may be enhanced when addressing the identified factors.

## Electronic supplementary material

Below is the link to the electronic supplementary material.

Additional file 1. Example questions from the semi-structured interview guides per perspective and tool.

Additional file 2. Results of the interviews from an educational perspective and professionals perspective per developed tool.

## Data Availability

The datasets used and analyzed during the current study are available from the corresponding author on reasonable request.

## List of abbreviations

OP: Occupational physician
IP: Insurance physician
COREQ: Consolidated Criteria for Reporting Qualitative research
WMO: Dutch Medical Research Involving Human Subjects Act
PA: Participatory Approach
