# Supplementary material for: Improving person-centered occupational health care for workers with chronic health conditions: a feasibility study
Source: BMC Med Educ. 2023 Apr 7;23:224. doi: 10.1186/s12909-023-04141-3 (PMC10082533; doi:10.1186/s12909-023-04141-3)
Supplement: Supplementary file 1 — Additional file 1. Example questions from the semi-structured interview guides per perspective and tool. [file 12909_2023_4141_MOESM1_ESM.docx]

Additional file 1. Example questions from the semi-structured interview guides per perspective and tool.

*1) Strengthening self-control of workers with chronic health conditions*

Educational perspective:

- Is there anything missing in the formulation of the learning objectives?
- Do the learning objectives fit in with the postgraduate training of OPs and IPs?
- Is there a need for the training in postgraduate training/continuing education?
- What are the reasons why the training does not match the learning objectives? Which elements make the training not appropriate to the learning objectives?
- To what extent can the training be integrated into the existing educational structures? Are adjustments necessary? If yes, what adjustments are needed?
- What is the need with regard the design of the training?
- How much time is required to prepare the training appropriately to fit the current educational structures?
- What resources (such as trainers/teachers, costs for the use of teaching rooms, costs for accreditation of continuing education) are required to provide the training?
- Is there another form of education through which the training can be integrated into the existing educational structure?
- What additional material is needed to integrate the training into the current educational structures?

Professional perspective:

- How do you look back to the participation in the training?
- What are the most important reasons why application of the acquired knowledge was not successful?
- Could the researchers of the project have done anything differently?
- Did you miss anything during the training?
- How does an organization have to look like to successfully apply the acquired knowledge?
- Did the training meet your expectations?

*2) Involving person-related factors (cognitions and perceptions) in the occupational health management and work disability assessment*

Educational perspective:

- Is there anything missing in the formulation of the learning objectives?
- Do the learning objectives fit in with the postgraduate training of OPs and IPs?
- Is there a need for the training in postgraduate training/continuing education?
- What are the reasons why the training does not match the learning objectives? Which elements make the training not appropriate to the learning objectives?
- To what extent can the training be integrated into the existing educational structures? Are adjustments necessary? If yes, what adjustments are needed?
- What is the need with regard the design of the training?
- How much time is required to prepare the training appropriately to fit the current educational structures?
- What resources (such as trainers/teachers, costs for the use of teaching rooms, costs for accreditation of continuing education) are required to provide the training?
- Is there another form of education through which the training can be integrated into the existing educational structure?
- What additional material is needed to integrate the training into the current educational structures?

Professional perspective:

- Are there elements of the training that need to be changed to make it easier to apply the acquired skills in practice?
- Are there things that make it difficult to apply the acquired skills?
- Do you have sufficient opportunities to apply the acquired skills in practice?
- The training is currently developed for both OPs and IPs together, does the content of the training have to be adapted per target group?
- Are there things that make it difficult to use the tool in practice?
- Do you think there are parts of the tool that need to be changed?
- To what extend can it be ensured that the tool can be used during consultation hour?
- How can the use of the tool be stimulated in practice?

*3) Involving significant others in the work re-integration process of workers with a chronic disease*

Educational perspective:

- Is there anything missing in the formulation of the learning objectives?
- Do the learning objectives fit in with the postgraduate training of OPs and IPs?
- Is there a need for the e-learning in postgraduate training/continuing education?
- What are the reasons why the e-learning course does not match the learning objectives? Which elements make the e-learning course not appropriate to the learning objectives?
- To what extent can the e-learning course be integrated into the existing educational structures? Are adjustments necessary? If yes, what adjustments are needed?
- What is the need with regard the design of the e-learning course?
- How much time is required to prepare the e-learning course appropriately to fit the current educational structures?
- What resources (such as trainers/teachers, costs for the use of teaching rooms, costs for accreditation of continuing education) are required to provide the e-learning course?
- Is there another form of education through which the e-learning course can be integrated into the existing educational structure?
- What additional material is needed to integrate the e-learning course into the current educational structures?
- How can the acquired knowledge be tested within existing educational structures?

Professional perspective:

- How did you experience the teaching method of an e-learning training compared to a traditional face-to-face teaching method?
- Is additional face-to-face teaching necessary for practical practice of the acquired knowledge?
- Are there elements of the training that need to be changed to make it easier to apply the acquired skills in practice?
- Are there things that make it difficult to apply the acquired skills?
- Do you have sufficient opportunities to apply the acquired skills in practice?
- What has changed in your behavior with regard to involving significant others since following the e-learning training?
- The training is currently developed for both OPs and IPs together, does the content of the training have to be adapted per target group?
- Are there things that make it difficult to use the tool in practice?
- Do you feel that the knowledge and skills acquired during the e-learning training are of added-value during your consultation hour?
- Do you think there are parts of the e-learning training that need to be changed?
- At the end of the e-learning, you will have access to materials (the tool): Are there materials that you have not used in practice?
- What makes it difficult to apply the skills and/or materials in practice?
- What can help to apply the acquired knowledge, skills and materials in practice?
