# Supplementary material for: Improving person-centered occupational health care for workers with chronic health conditions: a feasibility study
Source: BMC Med Educ. 2023 Apr 7;23:224. doi: 10.1186/s12909-023-04141-3 (PMC10082533; doi:10.1186/s12909-023-04141-3)
Supplement: Supplementary file 2 — Additional file 2. Results of the interviews from an educational perspective and professionals perspective per developed tool. [file 12909_2023_4141_MOESM2_ESM.docx]

Additional file 2. Results of the interviews from an educational perspective and professionals perspective per developed tool.

| **Bowen et al. focus area** | **Outcome** | **Category** | **Feasibility factor** | **Representative quote** |
| --- | --- | --- | --- | --- |
| **Project 1) Strengthening self-control of workers with chronic health conditions** | | | | |
| *1) Educational perspective* | | | | |
| Implementation | Factors affecting implementation ease or difficulty | Factors concerning the training | Explore possibility of conversion into an e-learning | P12: “I think, you need someone in the educational team for that [create an e-learning version]. […] I think, there first should be a wish from the educational team and then educationalists would start thinking about how you can create an e-learning from it [the current training].” |
|  |  |  | Offering an online version of the training | P15: “Just so you offer it online, in Zoom or something […].” |
|  |  | Factors concerning the organization of the training | Offer a train-the-trainer course | P12: “[…], so maybe you should use a train-the-trainer approach to make clear what your message is and it is really embedded in the educational program as intended.” |
|  |  |  | Hiring guest teachers is common practice | P13: “[…], we are used to hiring guest teachers who are expert on the subject for elective courses. So, we should also explore that for this case [the training] whether one of your people [from the research project] is willing to be a guest teacher.” |
|  |  |  | Coordinate with executive education manager | P13: “I am not the resident training director, I have no control over that.” |
|  |  |  | Fit training in time schedule used by educational institution | P15: “If you indicate how much time you need, that should be incorporated into the schedule.” |
| Practicality | Positive/negative effects on target participants | Added-value for participants | Clearly explain the added-value for OPs | P12: “OPs are very pragmatic people. They think: ‘Okay, I put time and money into this and I receive accreditation points, but what does it add for me in practice? How am I going to do my job better after I followed the training?’ If you can get that clear, I think, there is in interest in that [to follow the training].” |
|  | Cost analysis | Costs for organization of the training | Rental costs of training facility | P16: “The rental costs for the building […] are the highest.” |
|  |  |  | Costs for accreditation of training | P12: “[…] accreditation points must be awarded […]. Otherwise, nobody will come. They [registered OP/IP] only do this [follow the training] if it is accredited.” |
|  |  |  | Yields professional education points in postgraduate medical education without additional costs | P12: “[…] if we include it [the training] in the regular specialist training program, they will simply receive professional education points for that.” |
| Integration | Perceived fit with infrastructure | Suitability within educational structures | Not suitable for in-company training of insurance physicians | P14: “The training does not fit for the Social Security Agency, no.” |
|  |  |  | Not suitable for curriculum of postgraduate medical training for OP/IP | P13: “I don’t see, yet, that there is room for this [the training] in the curriculum of the specialist training program” |
|  |  |  | Added value of the training is evident | P12: “I think, this [training] adds value; it’s more specific. This [training] focuses not only skills of the OP during consultation, but rather on how the OP influences the work environment and that’s really the next step.” |
|  |  | Fit within the curriculum | Similar training already in elective course curriculum for OP/IP | P13: “We already have an elective day […] on this subject. Not exactly this variant, yet, but a day that is about the participatory approach.” |
|  |  |  | Training more suited for continuing education of registered OP/IP | P16: “I think it can be integrated. Then I go back to the question: ‘Is it more suited for for continuing education instead of the specialist training program?’. […] I think, it would be very good that we would at least get this for registered OPs, and possibly also IPs, but then I have to think about how.” |
|  |  |  | Requires effort to embed in curriculum | P13: “So, education that goes beyond that problem [of a sick worker], then you lose commitment and it requires work to embed in the curriculum, but we don’t have time for that.” |
|  |  |  | Offer as refresher course for continuing professional development | P12: “[…] we also have refresher courses […]. These are for registered OPs who want to learn more about this and then you reach a very targeted group […] wanting to learn how to tackle this at an organizational level.” |
|  |  |  | Mainly suitable for embedding in education for OPs | P15: “In particular, I think, you are more looking into the field of OPs.” |
| *2) Practical perspective of professionals* | | | | |
| Implementation | Factors affecting implementation ease or difficulty | Factors concerning the training | Structure of the training program | P19: “How you presented it [the tool] with first a presentation in PowerPoint and then the practical assignment […] fine, nothing to complain about.” |
|  |  |  | Affirm the need for an organization-intensive approach | P20: “[…] whether we should do something different in the training. Isn’t it [the application of the knowledge in practice] too intense to organize? An organization-intensive method […]. Are there organizations at all that want it [prevention] that way?” |
|  |  |  | Match with needs within organizations | P20: “I think, for the training we should assess more what they [the organization] are already doing. Is it [supportive work environment for workers with chronic conditions] an issue, what are they [the organization] already doing with it and what would they still need.” |
|  |  |  | Need for a script for OPs to use in the organization | P31: “[…] we now received all kinds of tools and documents, while I think, it would have helped if we had received some kind of script to use […].” |
|  |  |  | Use of actual cases from practice | P31: “But in the end you can also learn a lot about why it [application of the knowledge] doesn’t work within the organizations and that is perhaps something we should focus on first [in the training].” |
|  |  |  | Need for periodic reminder or refresher about the topic | P31: “So that means that if you don’t do it [apply the knowledge] and you want to bring it up again, you need an external reminder. Otherwise, it will be out of your system.” |
|  |  | Factors concerning the organization of the training | Involve higher management | P24: “It is also an organization where a lot of things are controlled from the headquarters and, yes, you may have to tackle it from a much higher perspective so you can say: ‘it is required from the headquarter’.” |
|  |  |  | Involving the researcher (of the project) | P20: “Yes, but it would be even stronger if you say: ‘in general we [the researchers] are coming to help the first time unless you have objections’.” |
|  |  |  | More information in advance | P21: “You should also make the time investment clear […]. I think, it might also help to see the program, to see that it is not even that big of a time investment.” |
|  |  |  | Involve account manager | P24: “No, I think in this organization […],I announced it to our account manager, the national account manager, who was very enthusiastic about it [the tool].” |
|  |  | Factors related to the dissemination | Embed in guideline | P24: “I had heard of it [the tool] and also read something about it, but now I immediately recognize it, because there is a new guideline now for working after cancer […].” |
|  |  | Personal factors | HR-officer too busy with other matters | P19: “It only gets worse. Now I’m in a reorganization again with outsourcing and stuff like that. My agenda is not getting better to be honest.” |
|  |  |  | High turnover of supervisors | P18: “And there are so many changes, which also means a lot of changes in managers, […] they also fly in and out again and, yes, then you have no continuity at all and, yes, that does not work well.” |
|  |  |  | Organizational support for the input from OP within company | P20: “If you apply this method [the tool], I think, that as an OP you are interested to bring in a bit of expertise. And that only works if you have support and […] if you already have the feeling that you can have a say in that [at the organization].” |
|  |  |  | Intrinsic motivation | P32: “I think, it just depends on being interested, first and wanting to know more about it [the topic on prevention]. I mean, in fact, it’s about being intrinsically motivated.” |
|  |  | Prerequisites for the application at organization | Requires organizational support | P19: “It must be accounted for support [from the organization] anyway, which means that there is no issues in the organizations, there are no problems in the organization, but that people say: ‘this is a great subject, we will work on it together, we will try our best’. While now you will have to work on it [the tool] yourself, but you will not get anyone enthusiastic for that [the tool].” |
|  |  |  | Recognition of importance for target group | P18: “Yes, just the sense of urgency that it is a group of people that we simply have to acknowledge [workers with a chronic condition].” |
|  |  |  | No sense of urgency | P23: “Well, the realization that people’s priorities and urgency within organization lie elsewhere.” |
|  |  |  | Presence of project leader | P20: “Yes, and the person who actually has do it [apply the tool], who has to lead that project, also needs to be present [during the training].” |
|  |  |  | Higher priority in organization with high absenteeism | P21: “Yes, if you have an absenteeism rate of 12%, then, yes, I think, then you see it [the need for the tool] differently.” |
|  |  | Impeding factors at organizational level | Other organizational matters with higher priority | P21: “You know, the organization is really busy and they [the organization] are indeed working on a large number of projects. I think, that was the factor.” |
|  |  |  | Financial situation of company | P19: “I also have to say that the business [in the organization] is nog going well now, far below expectations what they think should be done in terms of productivity […], profitability. People are just not satisfied […].” |
|  |  |  | Size of organization | P18: “Yes, it matters for what kind of organization it is. So, whether it is an internal health and safety service or a large company or a smaller company.” |
|  |  |  | Recent changes in organization | P24: “Yes, and in an organization in which a lot has changed and happened in recent years, I think, that would be a good reason to do this [apply the tool].” |
|  |  |  | Lack of staff | P24: “That something else is coming up that requires a lot of time. And it is also national. There are limited numbers of managers […].” |
|  |  |  | Need for clarity regarding required (time) investment | P20: “And it was also not estimated in advance. […] approximately how many meetings do you have to take into account, what is the impact, and for the OP, say, in terms of time investment, because they are also short on time these days.” |
|  |  |  | Own responsibility of organization | P21: “Actually, you also put it [the responsibility to make the tool a success] to the organizations themselves a bit.” |
|  |  |  | OP not involved in policy issues | P18: “Well, I’m just someone who carries out the tasks [to support sick-listed workers]. So, I’m not really involved in policy and I’m also not involved with those that are.” |
|  |  | Suitability | More suitable for self-employed OP | P18: “Yes, then that’s easy if you have something like that [being self-employed] […]. If you then have something like this [the tool] in your repertoire as an OP, yes. ” |
|  |  |  | Fits advisory role HR | P18: “In itself, I think, it was interesting and I think, it was nice and it was a tool that, I think, from an advisory role in the HR-setting, I think, it [the tool] did fit […].” |
|  |  |  | More suitable for individual guidance | P18: “Well, I had the idea that it [the tool] was more about how I, as an OP, could give guidance to people during consultation and maybe play an advisory role towards a manager or towards a department or an organization. So, I was thinking, more in that setting […] and not policy-wise.” |
|  |  |  | No uniform HR policy | P21: “So, it’s not like, that we’re going to just use this approach, because that [policy] can be different for each division and it can also be different for each HR officer. There is very little policy on paper.” |
|  |  |  | Being up-to-date | P20: “That’s often a great motivation, besides financially, of course, to do something. We don’t want to miss out and fall behind.” |
| Practicality | Ability of participants to carry out intervention activities | Personal influences | More support for unexperienced OP | P19: “I can imagine for someone [OP] who doesn’t know the topic, maybe a little more support is nice […].” |
|  | Cost analysis | Costs concerning the application in practice at an organizational level | Cost investment with unknown benefits | P20: “Well it also didn’t work on a small-scale, but maybe on a large-scale it would have succeeded, because then the time investment, so the total investment is the same, but perhaps much more profitable for an organization.” |
| Integration | Perceived fit with infrastructure | Integrate at organization | Include in company annual plan | P19: “It was not for nothing that we included it [the topic on chronic health conditions] in our annual plan that we adhere to […] with this organization […].” |
|  |  |  | Degree of professional flexibility of OP | P19: “I’m working at an internal [occupational health] service where they say, you have to ensure the health of the employees. Then your type of work gets a completely different content where I also get the freedom […]” |
|  | Perceived sustainability | Continuity | Continuity not guaranteed within the company | P18: “ […] there are so many changes going on, so you miss the continuity and you need that for such a process.” |
| **Project 2) Involving person-related factors (cognitions and perceptions) in the occupational health management and work disability assessment** | | | | |
| *1) Educational perspective* | | | | |
| Implementation | Factors affecting implementation ease or difficulty | Factors concerning the training | Possibility to differentiate focus for OP and IP | P13: “[…] you have put more emphasis on the analysis by the IP how to ask wat the problem is [for the worker with a chronic condition]. And for the OP more attention needs to be paid towards the intervention phase.” |
|  |  |  | Use of cases from practice | P16: “[…] it becomes a bit too much if we let groups go through the slides [of the training] from A to Z. So what we usually do is to make use of input from actor or use footage from an actual case.” |
|  |  |  | Sufficient interaction | P16: “And with those assignments, I think, it’s very smart to put the group to work, yes.” |
|  |  |  | Offering online version of the training | P16: “[…] I think a lot can be done online. With a bit of introduction by the trainer. And that a number of participants can then work online independently.” |
|  |  | Factors concerning the organization of the training | Offer a train-the trainer course | P15: “Well, I think, that the training can also be given by an institute trainer, if you prepare him properly.” |
|  |  |  | Coordinate with scientific associations of OP/IP | P13: “[…] change in the program [educational program] is also something that you should coordinate with the scientific associations.” |
|  |  |  | Coordinate with executive education managers | P13: “This is about communication. So, coordination would be necessary with the coordinator of the communication learning track.” |
|  |  |  | Fit training in time schedule used by educational institution | P16: “A half day [training] is appropriate. […] The evenings, that’s what I’m hesitant about. Our current target group is used to daytime education. But planning of half-days, yes, that fits within the existing structure of [organization].” |
|  |  |  | Involving the researcher (of the project) | P16: “[…] I think, that our institute trainer would also like it if one of the researchers is involved. Because otherwise, I think, we’ll soon get the comment like: ‘I’ll read through the sheets [the material] and I’ll figure out myself how I could do it [teach the material]’.” |
|  |  |  | Active involvement of the resident trainer | P13: “That would mean that you might also have to think about how to include the resident trainer in the training groups.” |
|  |  |  | Share training materials with educational institution | P13: “It would be valuable if you share the material and send the articles [scientific publications] you refer to as PDF to the teacher. Or at least the reference, so participants can easily find it.” |
|  |  |  | Make arrangements regarding ownership of the training | P15: “Well, if we incorporate it [the training] in our continuing education program, then it actually becomes our training. So then we have to see how we can do that […].” |
|  |  | Factors related to the dissemination | Use of trainers with adequate expertise | P16: “And quite frankly a number of institute trainers will say: ‘it [the topic] doesn’t fit my interest or expertise’. For whom would it be suited? So I do have a number of people in my head […].” |
|  |  |  | Contributions at conferences | P14: “And you can also test it at the OP conference days. That’s an annual conference. You could sell it well there. And then you can also see how it works with OPs. […] That’s also a great platform to draw attention.” |
|  |  | Personal factors | Need for actual training in own practice to develop skills | P13: “Let me say, what I can imagine is that if you offer this training, people have internalized it […], but are still hesitating about how they are going to apply this during their consultation. And that particularly applies for colleagues with relatively little work experience.” |
| Practicality | Ability of participants to carry out intervention activities | Personal influences | Difficult to transfer knowledge and skills into practice | P13: “What we notice in the training groups is that at least some of the participants say at the end of the day: ‘it was very useful, but I don’t see myself doing it [in practice] yet.’. And they, therefore, have difficulty translating it [the knowledge] into practice, into their own practice.” |
|  |  |  | Match with level of preexisting knowledge and skills of participants | P16: “Does the target audience know what they will be learning that day? Are they experienced IPs and OPs? Or are they residents who are at the beginning of their training? That means managing expectations in advance for both the teacher and participants.” |
|  | Cost analysis | Costs for the organization of the training | Rental costs of training facility | P16: “[…] we’re talking about staffing and renting spaces and IT facilities. […] Every training facility is the same, so the rent of the space.” |
|  |  |  | Costs of educational staff | P16: […] and staff, of course, is the biggest item.” |
|  |  |  | Costs of catering at training facility | P16: “With a whole day training, then we offer lunch and that kind of costs are added.” |
|  |  |  | Costs for professional education points of the training | P15: “[…] the moment we implement this [the training] in our resident training education, then the training is automatically awarded with professional education points . So, no, it doesn’t matter.” |
| Integration | Perceived fit with infrastructure | Fit within the curriculum | Possibilities for integration in curriculum postgraduate medical training OP/IP | P12: “I rather think that you could integrate this [training] with education that we already have and pay explicit attention to this [topic] by stating that in this case it’s about the chronically ill […].” |
|  |  |  | No unlimited place to embed new trainings in current curriculum | P12: “We need to comply with the national training plan from our professional scientific association about what should be included in our curriculum […]. […] So putting something new in […] you know, it takes a whole puzzle to figure it out. If this comes in [the program], what goes out?” |
| *2) Practical perspective of professionals* | | | | |
| Implementation | Factors affecting implementation ease or difficulty | Factors concerning the training | Mandatory homework assignment | P10: “Yes, it’s without obligations now, too casual.” |
|  |  |  | Use of actual cases from practice | P4: “Yes, I think, there could be a few more cases, which are more difficult.” |
|  |  |  | Need for follow-up training | P2: “Well, what I think could make a difference, because the training was only one time. So, that some sort of follow-up would be useful.” |
|  |  | Factors related to the tool | Layout needs to support usability of the tool | P5: “At some point I find it [the cognitions and perceptions] so connected that it can be taken together in the future to make it even simpler.” |
|  |  |  | Use of desk manual/summary as handy memory aid | P6: “[…] yes, I did that training then and I received a handy sheet. Now I’m going to put it in my laptop sleeve, so that it just goes to work with me. I think, a something to remember it helps […]” |
|  |  |  | Electronic pop-up window as reminder | P3: “Well, maybe with a pop-up like that on my computer. I have a kind of consultation hour format for myself with a number of topics that I want to discuss in any case.” |
|  |  | Factors related to the dissemination | Contributions at conferences | P5: “Yes, well we were talking about the training, but of course there are also various conferences every year. […] maybe there could be workshops or discussion groups organized that talk about this [the tool]. Yes, that leads to recognition […].” |
|  |  |  | Embedding in postgraduate medical training for resident OP/IP | P2: “I think, that it is very important. Of course, a whole group of young resident doctors is coming and already started. If they are educated with this, that’s very good, I think.” |
|  |  |  | Uptake in medical guideline for OP/IP | P1: “You could submit it at the quality agency of the scientific association where it [the tool] could become a part of an implementation training of a guideline.” |
|  |  | Personal factors | Need for external motivation | P3: “Well, I think, that there should be some kind of reward almost, that you notice that you receive some kind of result from it [following the training] […], that you get something in return.” |
|  |  |  | Need for sufficient time | P3: “Well, you always have to take that time yourself as an OP [during consultation]. […] I can take that [time] by doing longer consultation hours, that’s not the problem.” |
|  |  |  | Shared improvement of knowledge and expertise | P5: “It’s also about the experiences with the cases from practice and experience with the expertise of all those different colleagues. I think, that will ultimately lead to even more basis for [..] your tool.” |
| Practicality | Ability of participants to carry out intervention activities | Personal influences | Not knowing it by heart after training | P4: “Yes, […] when you are in a consultation hour, of course, you don’t have all those example questions [from the tool] in front of you anymore. So, you have to do it from your own memory. Apparently, the material hasn’t stuck yet so that I know it all by heart, so to speak.” |
| Integration | Perceived fit with infrastructure | Integrate in practice of OP/IP | Fits well into current consultation work method | P9: “The IP has some kind of list in mind that he works through [during consultation]. And some [points] are actually written out […], so that you are more aware […]. You can connect your material in one way or another.” |
| **Project 3) Involving significant others in the work re-integration process of workers with a chronic disease** | | | | |
| *1) Educational perspective* | | | | |
| Implementation | Factors affecting implementation ease or difficulty | Factors concerning the e-learning | Check if e-learning was completely followed | P15: “[…] you should do something as some kind of final assignment. Yes, that you can at least see that they [the students] looked at things [in the e-learning]. […] otherwise it would be too non-committal.” |
|  |  |  | Offering an online version of the training | P12: “You know, the entire area of education is now considering all kinds of online possibilities and e-learnings. So, this is, of course, very much welcomed at the moment. Personally, I think, that there is an increasing need for online education and e-learnings.” |
|  |  |  | Combining educational forms | P12: “Yes, what we ultimately want to achieve is some form of blended learning, in which physical education and online education and e-learning are all integrated with each other […].” |
|  |  |  | Integration with existing education material | P13: “[…] there is a good chance that some kind of hybrid product could be formed, a combination of what someone already developed plus some new elements from your tool based on research. I think, that’s easier to achieve than to delete things and replace them.” |
|  |  |  | Study credits in continuing education | P14: “And then they know for how many study points [they need to follow the e-learning], […] a course is accredited. And a certified OP/IP needs to get a certain amount of study points for continuing education per year. So that’s a good condition to get participants.” |
|  |  | Factors concerning the organization of the e-learning | Make arrangements regarding ownership of the e-learning | P16: “[…] in my practical experience, for example, […] an organization takes ownership and then it [the training] comes behind a pay-roll [not freely available].” |
|  |  |  | Combine developed trainings as coherent education program | P13: “Well, there are the training programs for education that are now presented as three separate pieces of education. But, of course, there should be coherence.” |
|  |  |  | Uptake as elective training in educational program | P14: “And if there is such a continuing education […] as a separate continuing education for people who have affinity with the topic or are interested themselves.” |
|  |  |  | Use of actual cases from practice for training | P13: “[…] the person that presents [in the e-learning] also presents from his own cases instead of the cases from someone else […].” |
|  |  |  | Involving the researcher (of the project) | P13: “In addition to teaching materials and underlying scientific publications, it would be good to also have a list of people [from the research] whom the teacher may contact to discuss things again.” |
|  |  |  | Curriculum planning is set well in advance | P13: “That means that if you come onto the market with a training package at any time, you can’t start with it in the three months after that. So, there’s a considerable lead time, because of the scheduling has to take place well in advance.” |
|  |  | Factors related to the dissemination | Use role models or frontrunners as trainers | P14: “I’m afraid if you don’t have someone who really advocates it [the training], then there’s a nice product sitting on a shelf somewhere and not much is done with it.” |
|  |  |  | Share material on website | P16: “[…] it would be very nice if you could indeed refer to either a website, or to a new website, […].” |
|  |  |  | Marketing the training through customer base | P14: “Look, that [organization], of course, has a large customer base with address files and that way you can sell a course.” |
|  |  | Personal factors | Resistance from educational trainers to incorporate new training material | P13: “You can also imagine that there is also a certain resistance among people who now teach about being chronically ill […]. […] and they first have to be convinced that this [e-learning] will be so much better.” |
|  |  |  | Requires competence level of registered OP/IP | P12: “It could be suitable for both groups [OP and IP] and I think, it fits better with continuing education, because this is something that is really a bit for more advanced OPs. […] I really see this as in-depth approach that you may not be ready for at the start of your training.” |
|  |  |  | Knowledge and skills relevant for GP | P12: “I think, it [the e-learning] is even more relevant for clinical doctors. So, I think, GPs, because as an OP, you rarely have family members come along [to the consultation].” |
|  |  |  | Necessary knowledge and skills for OP/IP | P15: “It [the knowledge from the e-learning course] is knowledge that you [OP or IP] must have, in my opinion.” |
| Practicality | Cost analysis | Costs for the organization of the e-learning | Costs for accreditation in case of refresher training for registered OP/IP | P14: “And you need to pay for that and then you get points for that [following the e-learning]. And you also need them [the points] for your re-registration as a doctor.” |
|  |  |  | Commercial market for continuing education for registered OP/IP | P14: “There is a commercial market beyond that. So, every IP, including me, and OP get e-mails from private companies […]. Well, there is a whole market for that to offer refresher courses for registered IPs and IPs sign-up for it.” |
| Integration | Perceived fit with infrastructure | Suitability within educational structures | Added-value of the e-learning is evident | P14: “With my many years of experience, I’m convinced of it [the e-learning]. I think, it’s a good thing that this [involving significant others] is getting some attention.” |
|  |  |  | Not suitable for core curriculum of postgraduate medical training for OP/IP | P14: “But, to incorporate it in the basic education package, that seems to be going a bit too far. Also, because you don’t have that kind of contact with people when reintegrating [a sick-listed worker].” |
|  |  |  | Suitable for postgraduate resident training and for continuing education | P15: “It [the e-learning] should really just be a standard part in the postgraduate resident training. But for those [registered OP/IP] who haven’t had it, yet, you could offer it [the e-learning] in continuing education.” |
|  |  |  | Impact on available time for consultation hours | P14: “[…] if you send all those doctors to a lesson with an e-learning, it will be at the expense of the number of consultations hours. […] that means that you have to take people out of production.” |
|  |  |  | Suitable for labour experts | P30: “So, I think, looking at labour experts, it will also apply to the [organization]. […] labour experts who are hired by private companies, for example, […] have a guiding role. […] So I find it very relevant for that population of occupational experts and actually find it more relevant than for the IPs.” |
|  |  | Fit within the curriculum | No flexibility to fit in in-company trainings | P14: “The [organization] is not flexible at all. It’s very big […] it always comes down to the production.” |
|  |  |  | Fit with learning goals of medical training OP/IP | P16: “Yes, it [the learning goals] would fit within the objectives.” |
|  |  |  | No unlimited place to embed new trainings in current curriculum | P14: “You can put it in the specialist training program within the [organization], but the curriculum is already overfull. Things [education] have to get out [the curriculum].” |
|  | Perceived sustainability | Continuity | Continuity after research project ends | P16: “Experiences show that after the completion of projects, sometimes, […], things come to a standstill.” |
| *2) Practical perspective of professionals* | | | | |
| Implementation | Factors affecting implementation ease or difficulty | Factors concerning the e-learning | Check if e-learning was completely followed | P28: “[…] with an e-learning if you do it […], you say, well everyone has to do that [the e-learning] within two weeks or a month.” |
|  |  |  | Appropriate form of education | P29: “Yes, actually very nice, because of course you can do it [the e-learning] in your own time, you can concentrate well, you know how long it takes, so you can plan well.” |
|  |  |  | Home assignments would facilitate use of the materials | P29: “That’s why I also said, if you could make one of the most important materials in such a way that you say: ‘do it as a homework or offer concrete assignments to use the material with two clients and see what the result is.” |
|  |  |  | Need for a refresher course | P30: “Yes, some kind of refresher things [courses]. […] I’ve never came across anything like that to be honest.” |
|  |  |  | Need for take-home message and repetition | P30: “When I go to a refresher course, then I always think to myself that I have to, I have to get about four or five learning points and more does not stick. […] You are in a refresher course all day long and you should actually have a few take-home messages that you can work with.” |
|  |  |  | Repetition of information within the e-learning | P25: “At a certain point [during the e-learning course], I thought: ‘oh, it’s going to be a lot of repetition’. But I also know that repeating is also good to understand the material. Otherwise it won’t stick.” |
|  |  |  | Keep the e-learning up-to-date | P29: “Maybe you’ll continue to evaluate and maybe add a few more things and stuff. So that the e-learning course can, of course, always be expanded further.” |
|  |  |  | Need for periodic reminder or refresher about the topic | P29: “[…] it [the knowledge] fades away after a while, doesn’t it? […] When you’ve gone through that process [following the e-learning], once a while, well every month or three months or certain moments you should have a reminder about: ‘you have these topics, have you actually discussed them in the past period, what have you done with it’. Just briefly that you’re reminded again.” |
|  |  |  | Different focus preferences for OPs/IPs | P25: “Look, within the occupational health group of OPs and IPs, you have a large common denominator, but a lot of individual variation. […] And that individual variation is colored […] by: where do you work, but also your areas of interest and your knowledge and skills.” |
|  |  |  | Length of the e-learning course | P25: “[…] what I remember about it [following the e-learning course]: I just went through it all the way and did everything and with great pleasure. […] it wasn’t like the overall length was too long or anything, […].” |
|  |  |  | Variation in teaching methods in the e-learning | P27: “What I remember is that it [the e-learning] was well put together. Because of the variation between theory and practical questions.” |
|  |  |  | Offer in a hybrid form | P25: “I think, you can also make a combination […] with e-learning, which also includes materials in which people [OPs/IPs] have to apply things themselves.” |
|  |  | Factors related to the tool | Direct digital results after filling out questionnaires | P30: “[…] if it’s the case that you can fill it [a questionnaire from the material] in and then get it back already filled in […], yes, then it could work.” |
|  |  |  | Integrate questionnaires in (IT-) systems of organization | P30: “[…] I imagine, that at a health and safety service there will be some kind of toolbox, I think, for OPs to use during their work. And you should actually see that you end up using the material from that toolbox.” |
|  |  |  | Use of desk manual/summary as handy memory aid | P29: “[…], we also have short summaries for standards and also those browsing systems have been developed that you can place on your desks […] to look up things a bit faster. Maybe also some kind of handout, very short, and maybe in collaboration with some learnings programs that you have to do.” |
|  |  |  | Offer additional material in accessible digital format | P28: “Just a PDF that I can send [to workers] is also nice. But then I would also like to have it in word. […] it’s easier to fill-in.” |
|  |  | Factors concerning the organization of the e-learning course | Promote the e-learning through scientific associations of OP/IP | P27: “But especially from the professional scientific association, I get [information] in all sorts of ways […]. It works, it keeps the subject alive in your head.” |
|  |  |  | Don’t make the e-learning course an obligation | P27: “Especially, don’t make it [following [the e-learning course] a standard policy. […] With us [OPs] it works that we take turns trying out something new [new working method] and make each other enthusiastic about it. But imposing something, we just don’t like that and we already have enough to deal with other requirements.” |
|  |  |  | Uptake as elective training in educational program | P26: “I would do that as an additional education.” |
|  |  | Factors related to the dissemination | Use role models or frontrunners as trainers | P27: “Success stories, yes, that’s personal. Success stories always work well.” |
|  |  |  | Embed in medical guideline for OP/IP | P27: “[…] the OPs guidelines are already very important. Then you should put this actually into a guideline.” |
|  |  | Personal factors |  |  |
|  |  |  | Sharing and learning with colleagues | P27: “Have worked for many years at [occupational health and safety service] […]. And there you also do it [following courses] together with several people from one organization. So you get the chance that they [colleagues] will talk about it at the coffee machine when we are back at the office. That’s how it [application of the tool] will come alive.” |
|  |  |  | Needed as basic knowledge for OP/IP | P25: “As an OP, I was already used to asking partners [to join the consultation hour]. But I do that particularly when a certain situation [with the worker] gets stuck or stagnation occurs, that I think: ‘yes, I just want that opinion of that partner, because he probably plays a role here.” |
|  |  |  | Necessary knowledge and skills for GP | P30: “[…] the doctor who sends people out with pills and then 60% won’t do it [take the pills], that’s what I mean. So it’s about effectiveness and efficiency of your consultation. And that [knowledge from the e-learning] should be applied for GPs.” |
|  |  |  | Resistance from educational trainers to incorporate new tool | P29: “I’m sure that my colleagues wouldn’t really start working with that [the tool] right away, to actually implement it in practice.” |
| Practicality | Positive/negative effects on target participants | Added-value for participants | Added-value of the e-learning | P25: “[…], but in the clinical work a new dimension has really been added […]. In particular the partners can play such a supportive role.” |
|  | Ability of participants to carry out intervention activities | Personal influences | Availability of tools/resources in own system | P30: “Yes, I have my own system. So what I did with the tools that came out [of the e-learning course], I put them in a folder and then I can choose from that folder.” |
|  |  |  | Responsibility of OP/IP to ask significant others to be present during a consultation | P26: “As far as I’m concerned that [involving the partner] is really something you as a doctor have to arrange and indicate when planning the consultation hour. And then it’s often no problem at all, it is simply scheduled and then someone gets an appointment and that’s it.” |
|  |  |  | Adopt new tools as part of the routine | P27: “[…] people must be prepared to change their own behavior. The OP must, of course, be prepared to accept it [the knowledge and tool] as a normal way of working. […] You just have to be willing to adjust your way of working a little bit.” |
|  |  |  | Theoretical knowledge increased awareness | P28: “I really like it [the e-learning course], because it was a subject that made me aware that I didn’t do it [involve significant others] much in my consultation hour.” |
|  |  |  | Only use material that seems relevant | P25: “That’s, of course, very often the case with continuing education, that you take away what is important to you and try it out in practice again.” |
|  |  | External influences | Time during consultation hour | P26: “In general, I think, […] if you think that it’s a complex situation [with the worker], then you could have an extended file or request an extended consultation hour.” |
|  |  |  | Take privacy regulations into account | P29: “You have to be careful due to the General Data Protection Regulation that you don’t just call a worker’s partner without their knowledge, for example.” |
|  |  |  | Enough freedom/room to apply skills | P30: “I have that [freedom] per definition. But that’s because I’m self-employed and an employer who calls me says in a contract: ‘first, I want to know how long it takes you during a consultation hour’., then I say: ‘we are not signing a contract with each other. I don’t do that.’. I always take the time I need […], but I realize that this is not possible for an average OP.” |
|  |  |  | Not suitable for all situations during consultation | P29: “You also need to be aware of which case it [involving significant others] is really suitable for and whether you have time for it and where you want to put an emphasis on.” |
|  | Cost analysis | Costs concerning the organization of the training | Commercial market for continuing education | P27: “Yes, in itself, the refresher course is free. Then you have a somewhat higher chance that people will follow it [the e-learning course].” |
| Integration | Perceived fit with infrastructure | Suitability within educational structures | Suitable for other disciplines | P30: “I can imagine that you should also be able to give that e-learning to physiotherapists. […] in the world of physiotherapy, quite a few people come with less well-understood pain complaints. […] I think, it could definitely fit there.” |
|  |  |  | Suitable for postgraduate resident training | P28: “[…] Just offer it in the beginning of the resident training for OPs, I think it would be nice.” |
|  |  |  | Suitable for basic medical education | P30: “[…] I think, that because what I just said about the academic level, the scientific framework behind that [the e-learning] that it would be fine to do it in the curriculum of medical students and then more in the direction of social medicine.” |
|  |  |  | Offer as elective education | P25: “People just look at what they are interested in to further dive into.” |
|  |  |  | Not suitable for basic medical education | P27: “On the other hand, you shouldn’t overload the young doctors with all these extra things [e-learning].” |
|  |  | Fit within the curriculum | Fit with learning goals of resident training for OP/IP | P25: “We have a basic [educational] block of communication which already contains various elements and this would be a nice strengthening education into the topic for people [OPs/IPs] who are interested in that.” |
